# *Pseudomonas bijieensis* Strain XL17 within the *P. corrugata* Subgroup Producing 2,4-Diacetylphloroglucinol and Lipopeptides Controls Bacterial Canker and Gray Mold Pathogens of Kiwifruit

**DOI:** 10.3390/microorganisms10020425

**Published:** 2022-02-12

**Authors:** Md. Arshad Ali, Jinyan Luo, Temoor Ahmed, Jiannan Zhang, Ting Xie, Dejiang Dai, Jingyong Jiang, Jie Zhu, Sabry Hassan, Jamal A. Alorabi, Bin Li, Qianli An

**Affiliations:** 1 State Key Laboratory of Rice Biology, Ministry of Agriculture Key Laboratory of Molecular Biology of Crop Pathogens and Insect Pests, Key Laboratory of Biology of Crop Pathogens and Insects of Zhejiang Province, Institute of Biotechnology, College of Agricultural and Biotechnology, Zhejiang University, Hangzhou 310058, China; alibau201@gmail.com (M.A.A.); temoorahmed@zju.edu.cn (T.A.); 21916084@zju.edu.cn (J.Z.); 22016086@zju.edu.cn (T.X.); libin0571@zju.edu.cn (B.L.); 2 Department of Plant Quarantine, Shanghai Extension and Service Center of Agriculture Technology, Shanghai 201103, China; toyanzi@126.com; 3 Station for the Plant Protection & Quarantine and Control of Agrochemicals Zhejiang Province, Hangzhou 310004, China; 4 Taizhou Academy of Agricultural Sciences, Linhai 317000, China; jjy5971@163.com; 5 Wenzhou Station of Plant Protection, Soils and Fertilizers, Wenzhou 325000, China; jane1979888@126.com; 6 Department of Biology, College of Science, Taif University, P.O. Box 11099, Taif 21944, Saudi Arabia; hassan@tu.edu.sa (S.H.); dr.jamal.a@gmail.com (J.A.A.)

**Keywords:** biocontrol, *Pseudomonas syringae* pv. *actinidiae*, *Botrytis cinerea*, DAPG, cyclic lipopeptides

## Abstract

Kiwifruit worldwide suffers from the devastating diseases of bacterial canker caused by *Pseudomonas syringae* pv. *actinidiae* (Psa) and gray mold caused by *Botrytis cinerea*. Here, an endophytic bacterium XL17 isolated from a rape crown gall was screened out for its potent antagonistic activities against Psa and *B. cinerea*. Strain XL17 and its cell-free culture filtrate (CF) inhibited the growth of Psa and *B. cinerea*, Psa-associated leaf necrosis, and *B. cinerea*-associated kiwifruit necrosis. Electron microscopy showed that XL17 CF could damage the cell structures of Psa and *B. cinerea*. Genome-based taxonomy revealed that strain XL17 belongs to *Pseudomonas bijieensis* within the *P. corrugata* subgroup of the *P. fluorescens* species complex. Among the *P. corrugata* subgroup containing 31 genomospecies, the presence of the *phl* operon responsible for the biosynthesis of the phenolic polyketide 2,4-diacetylphloroglucinol (DAPG) and the absence of the lipopeptide/quorum sensing island can serve as the genetic marker for the determination of a plant-protection life style. HPLC detected DAPG in extracts from XL17 CF. MALDI-TOF-MS analysis revealed that strain XL17 produced cyclic lipopeptides of the viscosin family and orfamide family. Together, phenotypic, genomic, and metabolic analyses identified that *P. bijieensis* XL17 producing DAPG and cyclic lipopeptides can be used to control bacterial canker and gray mold pathogens of kiwifruit.

## 1. Introduction

The genus *Pseudomonas* is one of the most complex and diverse genera among Gram-negative bacteria and consists of more than 300 species described to date (https://lpsn.dsmz.de/genus/pseudomonas, accessed on 2 October 2021) [1]. *Pseudomonas* is ubiquitous in aquatic and terrestrial environments and in association with diverse hosts including plants and animals [1,2]. Among plant-associated *Pseudomonas*, both pathogenic and beneficial strains are reported in the same or different species. For example, the *P. syringae* species complex contains plant pathogens of a wide range of plant species [3] while in the *P. fluorescens* species complex, multiple species are recognized as pathogens (such as *P. corrugata* and *P. mediterranea*) and plant growth-promoting bacteria (such as *P. brassicacearum*, *P. chlororaphis*, and *P. protegens*) [2].

Kiwifruit (*Actinidia chinensis*) is native to China and is a nutrient-dense fruit becoming popular worldwide [4,5]. Kiwifruit is susceptible to various pathogens that cause diseases at different growth stages, as well as after harvest. The bacterial canker disease caused by *Pseudomonas syringae* pv. *actinidiae* (Psa) is the most prevalent and devastating pandemic disease of kiwifruit [6,7]. The symptoms include necrotic leaf spots surrounded by a chlorotic margin, twig die-back, blossom necrosis, reddening of the lenticels, and bleeding cankers on the trunk and the leader with a whitish to orange ooze [6]. The gray mold disease caused by *Botrytis cinerea* is the most prevalent and devastating postharvest disease of kiwifruit, accounting for up to 30% of total postharvest losses in extreme cases [8,9]. Gray mold generally starts at the stem end of kiwifruit and progress evenly toward the distal part. The infected areas of the fruit are darker than the healthy parts. The white or gray mycelia and spores may be seen in affected areas with dark green and water-soaked lesions in internal tissues [9].

No curative approaches have been developed for management of the two devastating diseases of kiwifruit [7,8]. Copper compounds (e.g., copper hydroxide and copper sulphate) and antibiotics (e.g., streptomycin and kasugamycin) have been used to control the bacterial canker disease with limited success [10,11]. Psa has developed resistance to both copper and streptomycin [10]. Likewise, *B. cinerea* has also developed resistance to fungicides along with the fungicide application to control the gray mold disease [12,13]. Moreover, continuous applications of the chemical pesticides contaminate fruits, threaten human health, and pollute environments [14]. Therefore, integrated managements for the sustainable production of kiwifruit are in progress, including the development of resistant cultivars and pollinators, optimization of cultural practices, equilibrated plant nutrition, precise scheduled spray treatments with effective and eco-friendly antimicrobial agents, compounds inducing plant systemic resistance, and biocontrol agents [6,8,14,15].

Psa can survive on the surface of plant organs including leaves, twigs, buds, flowers, and fruits [16,17] and enter the plant through natural openings (e.g., stomata and lenticels) or wounds (e.g., fruit abscission scars or fresh cuts) and undergo a transition from the epiphytic phase to the endophytic phase, then move in plant tissues via apoplast and to different organs preferentially through xylem [18,19,20,21]. *B. cinerea* can infect leaves, stems, flowers, and fruits and live as endophytes before causing disease [22].

Endophytes can colonize the same sites as pathogens and compete against pathogens for niches and nutrition, and may directly inhibit pathogen growth or induce plant defense to reduce pathogen infection, and thus can be effective biocontrol agents to control plant diseases [23]. To develop effective biocontrol agents with endophytes, we screened endophytic bacteria from various plants based on broad-spectrum antimicrobial activities [24]. We found a strain which was isolated from a surface-sterilized crown gall of a rape plant and showed exceptional antagonistic activities against Psa and multiple fungal pathogens including *B. cinerea*. Here, we identify this strain belonging to *P. bijieensis* within the *P. corrugata* subgroup of the *P. fluorescens* species complex and determine its biocontrol potentials and mechanisms against Psa and fungal pathogens.

## 2. Materials and Methods

### 2.1. Microbial Strains

Psa strain ML2-12 was isolated from a bacterial canker stem of a kiwifruit plant grown in Taizhou, China. *Botrytis cinerea* strain B05.10 is a haploid strain derived from the monoascospore isolate SAS56 from grapevine (*Vitis vinifera*) [25].

Bacterial strain XL17 was isolated from a surface-sterilized crown gall of a rape plant (*Brassica napus*) grown in Hangzhou, China. The crown gall was surface-sterilized by 70% ethanol for 1 min and 5% sodium hypochlorite for 5 min and washed with sterile water six times, then ground in 1 mL of sterile water. The homogenate was streaked on a modified yeast extract–mannitol agar (yeast extract 0.08 g, mannitol 1.0 g, K_2_HPO_4_ 0.25 g, KH_2_PO_4_ 0.25 g, MgSO_4_·7H_2_O 0.2 g, NaCl 0.1 g, agar 15 g per liter; pH 7.0) and incubated at 30 °C for 7 d. Bacterial colonies showing different morphologies were purified by streaking. Purified bacteria were cultured in LB medium (yeast extract 5 g, tryptone 10 g, and NaCl 10 g per liter; pH 7.0) and then preserved with 15% (*v*/*v*) glycerol at −80 °C.

### 2.2. Assays of Antimicrobial Activities for Strain XL17

Bacterial isolates from the surface-sterilized crown gall were screened against fungal pathogens by the bacteria-fungi confrontation assay on potato dextrose agar (PDA) as previously described [24]. Bacterial strains showing potent antifungal activity were selected and further screened for antibacterial activity against Psa by the overlay culture assay on LB agar [26]. Strain XL17 was screened out for its potent antagonistic activities against fungal pathogens and *Psa*.

Strain XL17 was cultured in LB broth at 30 °C for 48 h. The culture was adjusted to approximately 1 × 10^8^ colony forming unit (CFU)∙mL^−1^ with sterile ultrapure water and centrifugated at room temperature. The supernatant was filtered through a sterile 0.22 µm filter and used as the culture filtrate (CF). The CF was added into LB medium to a final concentration of 10%, 15%, and 20% (*v*/*v*).

The effect of CF on the growth of *B. cinerea* was examined in potato dextrose broth (PDB) (potato infusion 200 g, glucose 20 g per liter; pH 5.6) according to [27]. A 5 mm plug of *B. cinerea* was added into 50 mL of PDB containing 10%, 15%, or 20% (*v*/*v*) CF and kept at 28 °C for 7 d. Fungal mycelia were obtained after filtering with a filter paper (Sinopharm Chemical Reagent Co., Ltd., Shanghai, China) and dried in an oven at 65 °C to a constant weight. The experiment was done tree times with three replications for each treatment.

The effect of CF on the growth of Psa was examined in LB broth containing 10%, 15%, or 20% (*v*/*v*) of CF in a 96-well microplate (Corning-Costar Corp., Corning, NY, USA). Psa strain ML2-12 was cultured in liquid LB medium for 16 h; 10 µL of the Psa culture was added into each microplate well. LB broth containing CF (200 µL) was added into microplate wells. LB broth without CF was used as control. The microplate was incubated at 30 °C for 24 h and the optical density at 600 nm (OD600) was measured with a SpectraMax spectrophotometer (Molecular Devices, Sunnyvale, CA, USA). The experiment was done three times with three replications for each treatment.

### 2.3. Scanning and Transmission Electron Microscopy on the Structure of Psa and B. cinerea

Blocks of 7-day-old *B. cinerea* mycelia grown on PDA alone or PDA containing 20% CF of strain XL17 were prepared for scanning electron microscopy (SEM) and transmission electron microscopy (TEM) as previously described [24]. Psa grown for 24 h in LB broth or LB containing 20% CF of strain XL17 was precipitated by centrifugation and then prepared for SEM and TEM as previously described [24].

### 2.4. Leaf Assay of Control Efficacy on Psa

The biocontrol potentials of XL17 and its CF against Psa were tested with detached leaves. Healthy leaves of similar sizes of kiwifruit plants (*Actinidia chinensis* var. *deliciosa* ‘Hayward’) were collected from the orchard. Leaves were surface sterilized by immersing in 70% ethanol for 1 min, 1.5% sodium hypochlorite for 1 min, and washing with sterile water six times [18]. The sterilized leaves were air dried inside a clean bench and the backside was cross marked with a sterilized needle. Each leaf was kept on a moistened filter paper inside a 15 cm petri dish. A drop (20 µL) of XL17 culture (1 × 10^8^ CFU∙mL^−1^), XL17 CF (10%, 15%, or 20% in sterile water), streptomycin sulphate (65 µg∙mL^−1^) [28], or sterile water (control) was dropped on the cross marked area and air-dried. Then, 20 µL of Psa culture (1 × 10^8^ CFU∙mL^−1^) was dropped on the same cross marked area. Only sterile water was used as negative control while Psa culture with sterile water was used as positive control. The leaves were kept in a growth chamber under 28 °C, a 16 h light (210 µmol/m^2^·s) and 8 h dark photoperiod, and 75% relative humidity for 10 d. The leaf necrotic area was measured and the percentage of inhibition was calculated compared to the control [29]. The experiment was done three times with three replications for each treatment.

### 2.5. Fruit Assay of Control Efficacy on B. cinerea

The biocontrol potentials of XL17 and its CF against *B. cinerea* were tested with fruits. Matured, uniform, healthy green-fleshed kiwifruits (*Actinidia chinensis* var. *deliciosa ‘Hayward’*) were bought from a supermarket (Walmart, Hangzhou, China). All fruits were surface sterilized by immersing in 2% (*v*/*v*) sodium hypochlorite for 2 min and washing with sterile water and then were air-dried inside a clean bench [30]. A 3 mm deep × 3 mm wide wound was made using a sterile needle on one side of each kiwifruit. A drop (20 µL) of XL17 culture (1 × 10^8^ CFU∙mL^−1^), XL17 CF (10%, 15%, or 20% in sterile water), difenoconazole (11.99 µg∙mL^−1^) [31], or sterile water (control) was dropped to the wound and air-dried. A 5 mm mycelial plug of *B. cinerea* was attached on the wound site of each fruit. Then, the fruits were kept on a moistened filter paper in a sterile plastic box (16 cm × 10 cm × 7 cm). The boxes were sealed with parafilm and incubated at 28 °C in the dark for 7 d. A sterile PDA plug with sterile water was used as negative control and a mycelial plug of *B. cinerea* with sterile water was used as positive control. Lesion area was measured and the percentage of lesion inhibition was calculated compared to the control. The experiment was done three times with three replications for each treatment.

### 2.6. Analysis of 16S rRNA Gene Sequences

The 16S rRNA gene sequence of strain XL17 was amplified from a colony by PCR using primers 27F (5′-AGAGTTTGATCCTGGCTCAG-3′) and 1492R (5′-GGTTACCTTGTTACGACTT-3′) as previously described [29]. The amplicon was sequenced using the Sanger method and a 1411 bp sequence was obtained and identified using the EzBioCloud identification service (https://www.ezbiocloud.net/identify, accessed on 4 October 2021). The 16S rRNA gene sequences of strain XL17 and type strains of closely-related *Pseudomonas* species were aligned using the MUSCLE program integrated in the MEGA5 software [32]. After eliminating positions containing gaps and missing nucleotides at both ends of the aligned sequences, 1405 final aligned nucleotides were constructed to a phylogenetic tree using the maximum likelihood method based on the Tamura–Nei model and Gamma-distributed with invariant sites for evolutionary rates and patterns.

### 2.7. Genome Sequencing and Assembly

The genomic DNA of strain XL17 was extracted using the SDS method [33] and quantified by a Qubit^®^ 2.0 Fluorometer (Thermo Scientific, Waltham, MA, USA). A 350 bp insert library was generated from 1 μg of DNA using a NEBNext^®^ Ultra™ DNA Library Prep Kit (New England BioLabs, Ipswich, MA, USA) and sequenced using an Illumina NovaSeq PE150 platform at the Beijing Novogene Bioinformatics Technology Co., Ltd. (Beijing, China). Low-quality reads in raw data containing low-quality bases (mass value ≤ 20) over 40%, N over 10%, or overlap with adapter sequences (length ≥ 15 bp, mismatch ≤ 3 bp) were removed by quality control using Readfq version 10. All good-quality paired-end reads (1277 Mb) of about 180-fold coverage were assembled using SOAPdenovo version 2.04 [34], SPAdes version 3.11.1 [35], and ABySS version 2.0.2 [36]. The assembly results were integrated using CISA version 4.0 [37] into the least 26 scaffolds with an N50 length of 771,204 bp. The resultinh draft genome contains 6,841,285 bp and has a G + C content of 60.84%. The draft genome sequence has been deposited at DDBJ/EMBL/GenBank under the accession no. JAJQKS000000000 and is annotated by the NCBI Prokaryotic Genome Annotation Pipeline [38].

### 2.8. Genome Relatedness Analysis

The digital DNA–DNA Hybridization (dDDH) value between pair genomes among strain XL17, strains showing the phylogeny of the 16S rRNA gene identical to strain XL17, and representative strains of species-level genomospecies within the *P. corrugata* subgroup was calculated using the Genome-to-Genome Distance Calculator (http://ggdc.dsmz.de/distcalc2.php, accessed on 6 October 2021) with Formula 2; a dDDH value of 70% was used as the threshold for species delimitation [39].

### 2.9. Genomic Analyses

The draft genome sequence of strain XL17 was annotated and amino acid sequences were predicted using the online platform RAST (Rapid Annotation using Subsystem Technology) version 2.0 (http://rast.nmpdr.org/, accessed on 8 October 2021). Gene clusters for the biosynthesis of secondary metabolites were found using the antiSMASH 6.0 pipeline with relaxed detection strictness (https://antismash.secondarymetabolites.org/, accessed on 10 October 2021) [40].

### 2.10. Phylogenomic Analysis of the Pseudomonas Corrugata Subgroup

Whole genome sequences (WGSs) of strain XL17, strains showing the phylogeny of the 16S rRNA gene identical to strain XL17, and representative strains of species-level genomospecies within the *P. corrugata* subgroup were selected for phylogenomic analyses (Table S1). These WGSs were annotated using RAST for pan-genome analysis. The phylogenomic tree was constructed for the *P. corrugata* subgroup based on the proteins encoded by their core genomes. *P. aeruginosa* DSM 50071^T^ was selected as the outgroup. Orthologous clusters of proteins were analyzed and output by running the pan-genomes analysis pipeline [41]. Core proteins were determined by a BLAST E-value < 1e^−10^, sequence identity > 50%, aligned sequence length coverage > 50%, and score > 40. The amino acid sequences from 1663 concatenated core proteins were concatenated and aligned using MAFFT version 5 [42]. The poorly aligned positions and excessively divergent regions were trimmed using GBlock 0.91b [43]. The resulting 509,085 amino acids were used to generate a maximum likelihood tree with the JTT + F + I + G4 model using the IQ-TREE version 2.1.2 [44]. The phylogenomic tree was displayed using the online tool iTOL version 5 [45].

### 2.11. Determination of 2,4-Diacetylphloroglucinol (DAPG) Produced from XL17

The presence of the *phl* operon (*phlABCD*) for biosynthesis of the phenolic polyketide DAPG was detected by PCR amplification of *phlD* partial sequences (about 745 bp) with the primers Phl2a (5′-GAGGACGTCGAAGACCACCA-3′) and Phl2b (5′-ACCGCAGCATCGTGTATGAG-3′) [46]. PCR was performed with the 2 × TSINGKE Master Mix (TsingKe Biological Technology, Beijing, China) and pre-denaturation at 94 °C for 3 min, 30 cycles at 94 °C for 1 min, 62 °C for 1 min, and 72 °C for 1 min, with the final extension at 72 °C for 3 min. The amplicon was sequenced using the Sanger method and identified by BLAST search in the NCBI database and submitted in GenBank under the accession no. MW851288. Phylogenetic analysis on the complete *phlD* sequences (1050 positions) from the WGS of strain XL17 and reference strains within the *P. corrugata* subgroup was performed using the MEGA5 software [32] with the maximum likelihood method based on the Tamura–Nei model and Gamma-distributed for evolutionary rates and patterns.

To identify DAPG produced by strain XL17, organic compounds were extracted from XL17 CF. Strain XL17 was cultured at 30 °C and 200 rpm for 48 h; 40 mL of the culture was acidified with 440 µL of 10% trifluoroacetic acid (TFA) to pH 2.0, and then was extracted twice with 100 mL of ethyl acetate. The extracted solution was evaporated with a rotary evaporator. The resulting dry extract was suspended in 5 mL of 35% acetonitrile with 0.1% TFA and then filtered through a 0.22 µm filter. Organic compounds were detected using an Agilent 1290 UHPLC system (Agilent Technologies, Santa Clara, CA, USA). An HPLC diode array detector was programmed to record absorbance at 270 and 310 nm. Standard DAPG solution (10 µg∙mL^−1^) was prepared with 35% acetonitrile and 0.1% TFA.

### 2.12. Detection of Lipopeptides Produced from XL17 Cells

Lipopeptides produced by a colony of strain XL17 were detected by matrix-assisted laser desorption ionization–time of flight mass spectrometry (MALDI–TOF–MS) as previously described [24].

### 2.13. Detection of Fluorescent Pyoverdine Siderophore

Fluorescent pyoverdine siderophore produced from XL17 colonies grown on King’s B medium was detected under UV light [47]. Strain XL17 was grown in LB broth at 30 °C for 48 h. The culture was washed with sterile water and diluted to 1 × 10^8^ CFU∙mL^−1^ and 5 µL of the diluted culture was dropped at the center of a King’s B agar plate. The culture was further diluted to 1 × 10^4^ CFU∙mL^−1^ and 50 µL of the diluted culture was spread on a King’s B agar plate. The plates were incubated at 30 °C for 48 h and were observed under UV light.

### 2.14. Detection of Hydrolytic Enzyme Activities

Enzyme activities of hydrolytic chitinases, β-1,3-glucanases, and proteases from strain XL17 was tested as previously described [24]. *Paenibacillus peoriae* CGMCC 1.3761^T^, which showed clear hydrolytic zones [24], was used as positive control.

### 2.15. Assay of Indole Acetic Acid (IAA) Production

IAA production by strain XL17 was determined using the colorimetric assay developed by Sarwar and Kremer [48] as previously described [24].

### 2.16. Detection of 1-Aminocyclopropane-1-Carboxylate Deaminase Activity

XL17 colonies grown on LB agar were streaked on solid nitrogen-free DF medium [49], DF medium with 3 mM 1-aminocyclopropane-1-carboxylic acid (ACC) as the sole nitrogen source, or DF medium with 1.5 mM (NH_4_)_2_SO_4_ as the sole nitrogen source, and allowed to grow for 2 d. Bacteria producing ACC deaminase can grow on the DF medium with ACC as the sole nitrogen source.

### 2.17. Assays of Toxic or Beneficial Potentials of Strain XL17 on Plants

Rice (*Oryzae sativa*) is one of the test crops for phytotoxicity assay recommended by the Organization for Economic Cooperation and Development [50]. Rice seeds (cv. II you 023) were surface sterilized by 3% sodium hypochlorite solution for 10 min and washed six times with sterile water. Rice seeds were soaked in sterile water overnight and then each seed in 1 mL of sterile water (control), XL17 culture, XL17 CF (10, 15 and 20%), streptomycin sulphate (65 µg∙mL^−1^), or difenoconazole (11.99 µg∙mL^−1^) for 4 h. Seeds (*n* = 20) were placed on a moistened sterile filter paper in a 15 mm Petri dish. The Petri dishes were kept in a growth chamber under 26 °C, a 16 h light and 8 h dark photoperiod, and 80% relative humidity for 7 d. The germination percentage, root length, shoot height, and dry weight of the rice seedlings were recorded. The experiments were done with three replicates.

To determine epiphytic root colonization by strain XL17, at 7 d after inoculation, bacteria adhering to roots were washed into 3 mL of sterile water and serially diluted; 100 µL of the bacterial suspensions were spread on LB agar. To determine endophytic colonization by strain XL17 in roots and stems, roots and stems were surface-sterilized by 70% ethanol for 1 min and 1% sodium hypochlorite for 2 min, and washed with sterile water six times. The sterilization efficacy was determined by no bacterial growth from the last-washed water. The surface-sterilized roots and stems were ground in sterile water. The homogenate suspensions were serially diluted; 100 µL of the diluted suspensions were spread on LB agar. After incubating the LB agar plates at 30 °C for 3 d, bacterial colonies showing the colony morphology of strain XL17 were counted. The experiments were done with three replicates.

### 2.18. Statistical Analysis

The SPSS 16.0 software (SPSS Inc. South Wacker Drive, Chicago, IL, USA) was used for statistical analysis of variance followed by post hoc multiple comparisons. The least significance difference test was performed to separate values of different treatments at *p* < 0.05. The values were the mean ± standard error of at least three replicates for each treatment.

## 3. Results

### 3.1. Strain XL17 Showed High Antimicrobial Activities against B. cinerea and Psa

**Figure 1:**
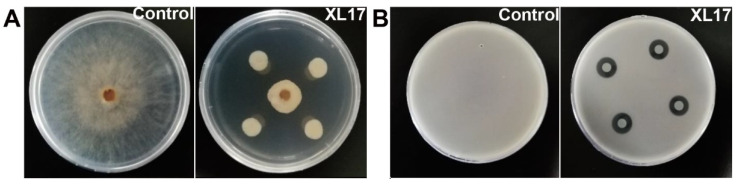
XL17 inhibits growth of gray mold *Botrytis cinerea* grown on potato dextrose agar for 7 d (**A**) and bacterial canker pathogen *Pseudomonas syringae* pv. *actinidiae* grown in LB agar for 3 d (**B**).

Strain XL17 inhibited radial growth of *B. cinerea* grown on PDA for 7 d up to 35 mm (Figure 1A) and inhibited growth of Psa grown in LB agar for 3 d to a clear zone of 15 mm diameter (Figure 1B).

**Figure 2:**
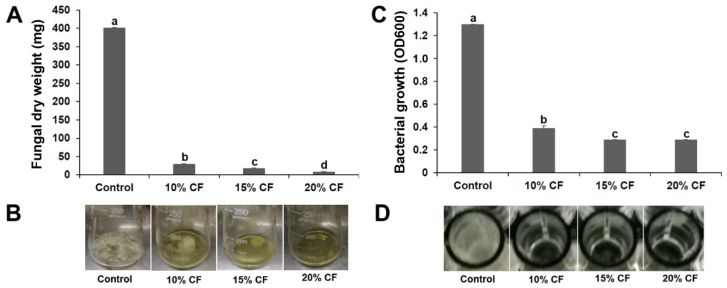
Culture filtrate (CF) of strain XL17 inhibits growth of *Botrytis cinerea* in potato dextrose broth (**A**,**B**) and *Pseudomonas syringae* pv. *actinidiae* in LB broth (**C**,**D**). Bacterial growth is indicated by optical density at 600 nm (OD600). The values are mean ± standard error of three replicates for each treatment. Different letters show significant difference at *p* < 0.05.

After 7 d growth in PDB containing 10%, 15%, or 20% CF of strain XL17, the dry weight of *B. cinerea* mycelia was 7.4%, 4.5%, and 2.1% of that in PDB without CF, respectively (Figure 2A,B).

After 24 h growth in LB containing 10%, 15%, or 20% CF of strain XL17 in microplate wells, the OD600 of Psa was 30%, 22%, and 22% of that in LB without CF, respectively (Figure 2C,D).

### 3.2. XL17 CF Damaged Psa Cells and B. cinerea Hyphae

**Figure 3:**
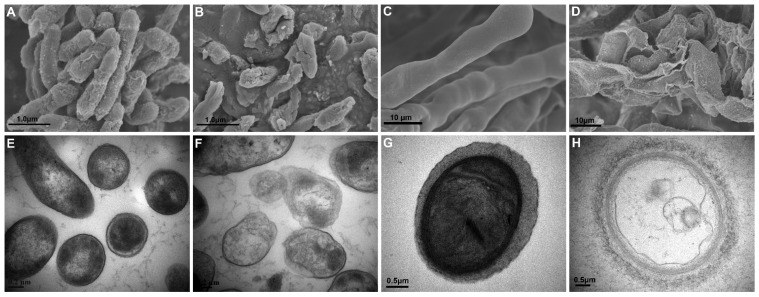
Structures of *Pseudomonas syringae* pv. *actinidiae* (Psa) cells and *Botrytis cinerea* hyphae shown by scanning electron microscopy (**A**–**D**) and transmission electron microscopy (**E**–**H**). Control Psa cells are intact (**A**,**E**) with electron-dense cell contents (**E**), whereas Psa cells grown with 20% culture filtrate of strain XL17 are broken and shrunken (**B**) and lost cell contents (**F**). Control hyphae with smooth and intact cells walls (**C**,**G**) contain electron-dense cell organelles and contents (**G**), whereas hyphae grown with 20% culture filtrate of strain XL17 are broken and fragmented (**D**) and lost cell contents (**H**).

SEM and TEM revealed cell damage of Psa and *B. cinerea* grown with XL17 CF. In contrast to the intact cell envelopes and condensed cell contents of control cells (Figure 3A,E), Psa grown with CF was broken and lost cell contents (Figure 3B,F). Likewise, control *B. cinerea* hyphae with smooth and intact cell walls contained electron-dense cell organelles and contents (Figure 3C,G), whereas hyphae grown with CF were broken and fragmented and lost cell contents (Figure 3D,H). XL17 CF contains antimicrobial agents which can breach the cell envelopes of the bacterial and fungal pathogens.

### 3.3. Strain XL17 and XL17 CF Reduced Leaf Necrosis Caused by Psa and Gray Mold Lesions in Kiwifruits

**Figure 4:**
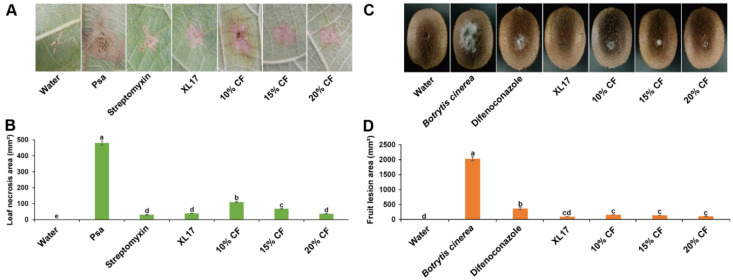
Strain XL17 and culture filtrate (CF) of strain XL17 inhibit necrosis in kiwifruit leaves caused by *Pseudomonas syringae* pv. *actinidiae* (Psa) after 10 d of wound inoculation (**A**,**B**) and lesions in kiwifruits caused by *Botrytis cinerea* after 7 d of wound inoculation (**C**,**D**). Leaf necrosis (**A**) caused by Psa under various treatments. Fruit lesions (**C**) caused by *B. cinerea* under various treatments. Leaf necrosis areas (**B**) and fruit lesion areas (**D**) are mean ± standard error of three replicates for each treatment. Different letters show significant difference at *p <* 0.05.

Psa caused necrosis in leaves after wounding inoculation. Streptomycin, XL17, and XL17 CF inhibited the necrosis. Streptomycin reduced 94% of the necrosis area while XL17 and 20% CF reduced 92% of the necrosis area after 10 d of the wounding inoculation (Figure 4A,B).

*B. cinerea* caused gray mold lesions in kiwifruits after wounding inoculation. Difenoconazole, XL17, and XL17 CF inhibited the lesions. Difenoconazole reduced 82% of the lesion area while XL17, 20%CF, 15% CF, and 10%CF reduced 95.5%, 94.6%, 93.0%, and 92.2%, respectively, of the lesion area after 7 d of the wounding inoculation (Figure 4C,D).

### 3.4. Strain XL17 Belongs to Pseudomonas bijieensis within the Pseudomonas corrugata Subgroup

**Figure 5:**
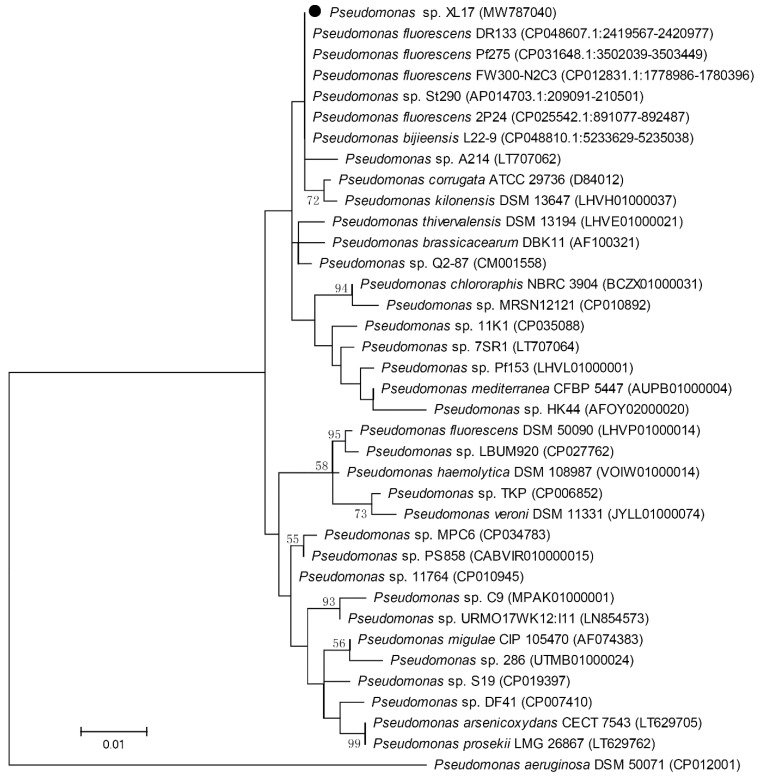
Maximum likelihood phylogenetic tree based on 16S rRNA gene sequences (1405 positions) of strain XL17 (●) and related *Pseudomonas* strains. *Pseudomonas aeruginosa* DSM 50071^T^ was used as outgroup. The percentages of replicate trees (>50%) in which the associated taxa clustered together in the bootstrap test (1000 replicates) are shown at the nodes. The GenBank accession numbers of the 16S rRNA gene sequences are indicated in brackets. The scale bar indicates 0.01 substitutions per site.

The 16S rRNA gene sequence of strain XL17 was amplified and a 1411 bp sequence was obtained. Blast search of the 1411 bp sequence showed that it is identical to the 16S rRNA gene sequences of *P. fluorescens* strains DR133, Pf275, and FW300-N2C3 and *Pseudomonas* sp. strain St290, one nucleotide different from that of *P. fluorescens* strain 2P24, and two nucleotides different from that of *P. bijieensis* type strain L22-9^T^. The phylogenetic tree constructed based on 1405 aligned positions of the 16S rRNA gene sequences showed identical phylogeny of strains XL17, DR133, Pf275, St290, L22-9^T^, 2P24, and FW300-N2C3 neighboring to *P. corrugata* (Figure 5). Based on 16S rRNA gene sequences, strain XL17 cannot be classified to a species but was classified to the *P. corrugata* subgroup.

To clarify the taxonomy status of strain XL17, we used the Illumina platform to sequence the WGS of strain XL17 and obtained a draft genome (accession no. JAJQKS000000000). Genome relatedness analysis showed that strains XL17, DR133, Pf275, St290, and 43MFCvi1.1 and *P. bijieensis* L22-9^T^ share dDDH similarities higher than 90% (Table S1) and thus belong to *P. bijieensis*. Strain 2P24 shares the highest dDDH similarities (about 62%) to *P. bijieensis* among all the genomes released in the NCBI genome database and thus represents a novel genomospecies most close to *P. bijieensis*. Strains FW300-N2C3, MPBD7-1, FW305-28, and PDM06 share dDDH similarities higher than 80% and belong to a same species; they share dDDH similarities lower than 70% to other genomes (Table S1) and thus represent a novel genomospecies. *P. bijieensis* is the species-level cluster (genomospecies) 18 of the *P. corrugata* subgroup within the *P. fluorescens* species complex while strain 2P24 represents genomospecies 17 and strain FW300-N2C3 represents genomospecies 22 [2] (Table S1).

### 3.5. Phylogeny and Genetic Markers Associated with Plant-Interaction Life Styles in the Pseudomonas corrugata Subgroup

**Figure 6:**
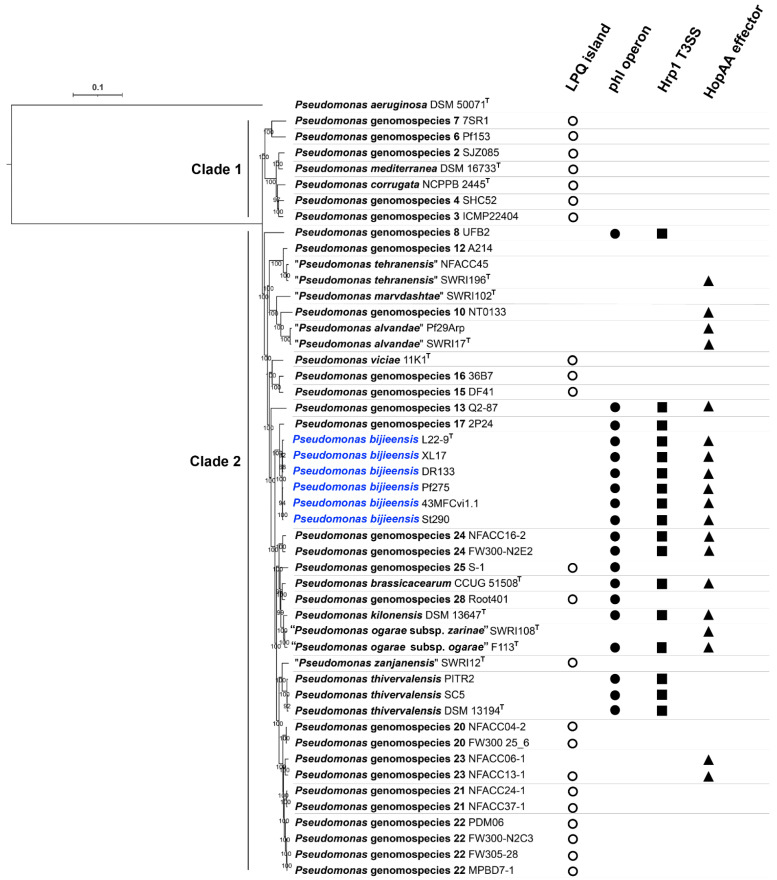
Phylogenomic tree based on concatenated 509,085 amino acid sequences of 1663 core proteins from strains within the *Pseudomonas corrugata* subgroup and the outgroup strain *P. aeruginosa* DSM 50071^T^. The maximum likelihood tree was generated using IQ-TREE 2.1.2 program with JTT + F + I + G4 model. Bootstrap values of 1000 tests are shown at the nodes. The scale bar indicates 0.1 substitutions per site. The *P. corrugata* subgroup consists of two monophyletic clades (Clade 1 and Clade 2). *Pseudomonas bijieensis* is highlighted in blue. Distribution of genetic markers LPQ island (**◯**), *phl* operon (●), Hrp1 T3SS (■), and HopAA effector (▲) associated with plant-interaction life styles are labelled after the strain holding the genetic markers.

Phylogenomic analysis based on 1663 core proteins sharing the *P. corrugata* subgroup and the outgroup *P. aeruginosa* DSM 50071^T^ showed that the *P. corrugata* subgroup contains two major monophyletic clades. Clade 1 contained *Pseudomonas* genomospecies 1 to 7 including *P. corrugata* (*Pseudomonas* genomospecies 5) and *P. mediterranea* (*Pseudomonas* genomospecies 1) [2]. All genomospecies within Clade 1 contain the lipopeptide/quorum sensing (LPQ) genomic island, which serves as a genetic marker for the plant-pathogenic life style of the *P. corrugata* subgroup [51], but not the *phl* operon (*phlACBD*) for DAPG biosynthesis, the gene cluster encoding a type III secretion system (T3SS) similar to the Hrp1 T3SS important for *P. syringae* virulence, and the single “orphaned” T3SS effector gene similar to the *P. syringae hopAA* gene (Figure 6). Clade 2 contained *Pseudomonas* genomospecies 8 to 29 [2], “*P. marvdashtae*” and “*P. zanjanensis*” [1] (Figure 6). Among the 24 genomospecies within the Clade 2, 10 genomospecies contain the LPQ island while 11 genomospecies contain the *phl* operon; only genomospecies 25 and 28 contain both the *phl* operon and LPQ island, while the 9 genomospecies containing the *phl* operon but no LPQ island contain the Hrp1 T3SS (Figure 6). The presence of *hopAA* is not congruent with the presence of the *phl* operon. Among the 11 genomospecies containing the *phl* operon, five genomospecies (*P. thivervalensis*, genomospecies 8, 17, 25, and 28) do not contain the *hopAA* gene (Figure 6). On the other hand, genomospecies containing the *hopAA* gene may not contain the *phl* operon, such as “*P. alvandae*”, genomospecies 10 and 23.

**Figure 7:**
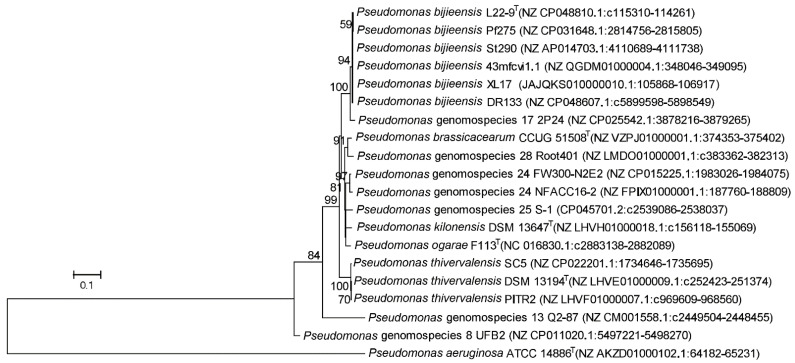
Maximum likelihood phylogenetic tree based on complete *phlD* gene sequences (1050 positions) encoding type III polyketide synthase of strains within the *Pseudomonas corrugata* subgroup. *P. aeruginosa* ATCC 14886^T^ was used as outgroup. The percentages of replicate trees (>50%) in which the associated taxa clustered together in the bootstrap test (1000 replicates) are shown at the nodes. The scale bar indicates 0.1 substitutions per site. The *phlD* gene sequences were extracted from whole genome sequences whose accession numbers in GenBank are indicated in brackets.

The phylogeny of the *phlD* gene encoding the type III polyketide synthase (Figure 7) was congruent with the species phylogeny (Figure 6). Notably, the genomospecies 26 was designated as “*Pseudomonas ogarae*” with the type strain F113^T^ by Garrido-Sanz et al. [2] and later as “*Pseudomonas zarinae*” with the type strain SWRI108^T^ by Girard et al. [1]. The members of *P. ogarae* formed two phylogroups [2] represented by strain F113^T^ and strain SWRI108^T^ (Figure 6); the two phylogroups share dDDH similarities of 73.0–75.6% (Table S2) below the DDH threshold of 79–80% for subspecies delimitation [52] and can be differentiated by the presence (*phl*+) and absence (*phl*−) of the *phl* operon (Figure 6; Table S2), and thus can be divided into two subspecies named as *Pseudomonas ogarae* subsp. *ogarae* (*phl*+) and *Pseudomonas ogarae* subsp. *zarinae* (*phl*−).

### 3.6. Genes Associated with Plant-Interaction Life Style in Pseudomonas bijieensis and Strain 2P24

Six strains belonging to *P. bijieensis* and strain 2P24-represented *Pseudomonas* genomospecies 17 formed a branch within Clade 2 (Figure 6). The antiSMASH 6.0 pipeline with relaxed detection strictness identified 15–18 regions for the biosynthesis of secondary metabolites from the WGSs of *P. bijieensis* strains and 14 gene clusters from the WGS of strain 2P24 (Figure S1). *P. bijieensis* strains share 15 biosynthetic gene clusters. *P. bijieensis* and strain 2P24 share 13 biosynthetic gene clusters and show their close relation.

*P. bijieensis* and strain 2P24 contain multiple gene clusters for the biosynthesis of antimicrobial agents. They contain the gene cluster for DAPG biosynthesis, having 100% similarity to the reference gene cluster from *Pseudomonas* genomospecies 13 strain Q2-87 (Figure S1). They contain the gene cluster for aryl polyene (APE Vf) biosynthesis, having 40% similarity to the reference gene cluster from *Aliivibrio fischeri* strain ES114 (Figure S1). They contain the gene cluster (Region 5.1 in strain XL17; Region 8 in strain 2P24) for the biosynthesis of a linear lipopeptide (Val-Ala-Gln-Ala-Val-Ala-Pro-Thr), having 8% similarity to the reference gene cluster for the biosynthesis of the cyclic lipotetradecapeptide entolysin from *P. entomophila* strain L48 and 17% (*P. bijieensis*) or 23% (strain 2P24) similarity to the reference gene cluster for the cyclic lipopeptide syringomycin from *P. syringae* strain B728a (Figure S1). This lipopeptide cluster encodes non-ribosomal peptide synthetase (NRPS)/polyketide synthase (PKS) (accession no. WP_232200821 and WP_232200822) similar to the cyclic lipopeptide syringopeptin NRPS (accession no. WP_161421051, identity about 48%), massetolide NRPS (accession no. ABH06368.2, identity about 42%), and orfamide NRPS (accession no. AAY91420, identity about 43%). These three gene clusters for the biosynthesis of DAPG, aryl polyene, and the linear lipopeptide have been identified for contribution to the biocontrol activity of *P. bijieensis* Pf275 [47].

*P. bijieensis* and strain 2P24 contain two or three NRPS gene clusters related to the biosynthesis of fluorescent siderophore pyoverdine (Figure S1). Pyoverdine biosynthetic gene clusters are present in all genomospecies within Clade 2 and the genomospecies 6 and 7 within Clade 1 as previously shown (Supplementary File S7 in Garrido-Sanz et al. [2]). In *P. bijieensis* WGS, one pyoverdine biosynthetic gene cluster (Region 11 in strain Pf275; Region 20.1 connecting with Region 1.1 in strain XL17) (Figure S1) including *pvdD* encoding a NRPS/PKS (Pf275 PvdD accession no. WP_116833073; XL17 PvdD accession no. WP_232201727) has been identified for its contribution to the biocontrol activity of *P. bijieensis* Pf275 [47].

*P. bijieensis* and strain 2P24 contain two other NRPS gene clusters related to metallophore biosynthesis. One (Region 17.1 in strain XL17; Region 1 in strain 2P24) has a 37% similarity to the reference gene cluster for the biosynthesis of the metallophore fragin from *Burkholderia cenocepacia* strain H111 (Figure S1). The other (Region 5.3 connecting with Region 10.1 in strain XL17, Region 9 in strain Pf275, and Region 10 in strain 2P24) has a 7% similarity to the reference gene cluster for the biosynthesis of the siderophore crochelin A from *Azotobacter chroococcum* strain NCIMB 8003 (Figure S1); the NRPS/PKS (accession no. WP_116832671 in Pf275) encoded by this gene cluster shows identities about 32–35% to those for the biosynthesis of the linear lipopeptide (Val-Ala-Gln-Ala-Val-Ala-Pro-Thr) and identities about 35–37% to those for the biosynthesis of the cyclic lipopeptides syringopeptin, massetolide, and orfamide.

*P. bijieensis* and strain 2P24 contain a butyrolactone biosynthetic gene cluster (Region 5.2 in strain XL17; Region 9 in strain 2P24) (Figure S1) encoding polyketide synthase (accession no. WP_232200998 in strain XL17 and WP_134924657 in strain 2P24) and polyketide cyclase (accession no. WP_232200999 in strain XL17 and WP_134924658 in strain 2P24), which may be involved in the biosynthesis of cyclic polyketides.

*P. bijieensis* and strain 2P24 contain a beta-lactone biosynthetic gene cluster (Region 2.3 in strain XL17; Region 6 in strain 2P24) having 13% similarity to the reference gene cluster for the biosynthesis of the antifungal lipopeptide fengycin from *Bacillus velezensis* strain FZB42 (Figure S1).

*P. bijieensis* and strain 2P24 do not contain the LPQ island carrying genes for the production of cyclic lipopeptide phytotoxins (syringopeptin and syringomycin) and the acyl-homoserine lactone quorum-sensing system, which is responsible for the pathogenicity of certain species within the *P. corrugata* subgroup [51,53].

*P. bijieensis* and strain 2P24 contain *hcnBAC* genes encoding hydrogen cyanide synthase (XL17 HcnA accession no. WP_162893903, HcnB accession no. WP_176688071, HcnC accession no. WP_116832142), which are present in all genomospecies within the *P. corrugata* subgroup as previously shown (Supplementary File S7 in Garrido-Sanz et al. [2]).

*P. bijieensis* and strain 2P24 contain two types (Hrp1 and SPI-1) of T3SS [2,54,55]. *P. bijieensis* but not strain 2P24 contains the single “orphaned” T3SS effector gene *hopAA* (Figure 6).

*P. bijieensis* and strain 2P24 contain genes encoding 1-aminocyclopropane-1-carboxylate (ACC) deaminase (accession no. WP_109753447), which is present in 20 genomospecies within the *P. corrugata* subgroup as previously shown (Supplementary File S7 in Garrido-Sanz et al. [2]).

### 3.7. Strain XL17 Produced DAPG and Cyclic Lipopeptides of the Viscosin Family and Orfamide Family

**Figure 8:**
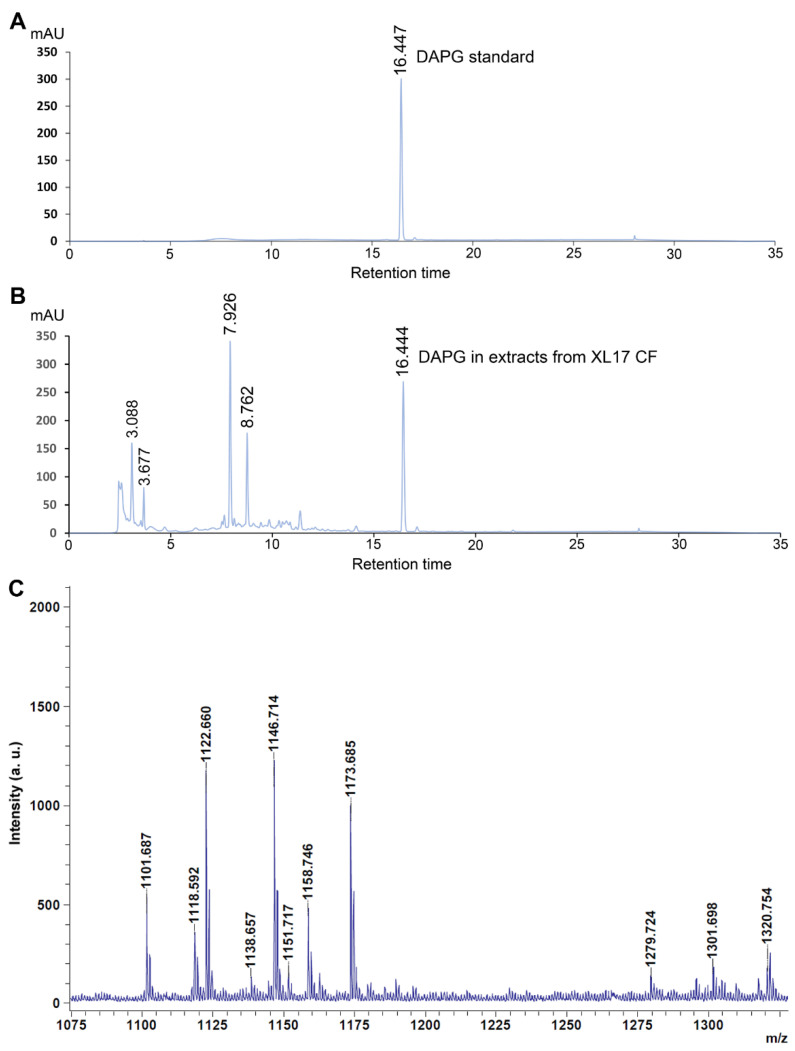
Strain XL17 produces polyketide 2,4-diacetylphloroglucinol (DAPG) and lipopeptides. (**A**) Standard DAPG (10 μg∙mL^−1^) detected by high-performance liquid chromatography (HPLC) has a retention time of 16.447. (**B**) DAPG detected from organic extracts of cell-free culture filtrate (CF) of strain XL17 grown in LB broth by the HPLC system has an almost identical retention time (16.444) as the standard DAPG. (**C**) Lipopeptides detected from a XL17 colony by MALDI-TOF-MS. Mass peaks ranging from 1110 to 1175 *m*/*z* and from 1250 to 1330 *m*/*z* indicate cyclic lipopeptides of viscosin family and orfamide family, respectively.

Organic compounds were extracted using ethyl acetate from acidified CF of strain XL17 at the stationary phase grown in LB broth and then determined by HPLC as well as the DAPG standard solution (10 μg∙mL^−1^). DAPG standard was detected at the retention time of 16.447 by the absorbance at 270 nm (Figure 8A). A component from the organic extract of XL17 CF showed an almost identical retention time (16.444) (Figure 8B) as the DAPG standard and thus was identified as DAPG.

From XL17 colonies, MALDI-TOF-MS detected cyclic lipopeptides of the viscosin family, including massetolides and viscosins with mass peaks ranging from 1110 to 1175 *m*/*z* [56,57,58], and the orfamide family, with mass peaks ranging from 1250 to 1330 *m*/*z* [59,60,61] (Figure 8C).

### 3.8. Strain XL17 Produced Pyoverdine, β-1, 3-Glucanase, and Protease

**Figure 9:**
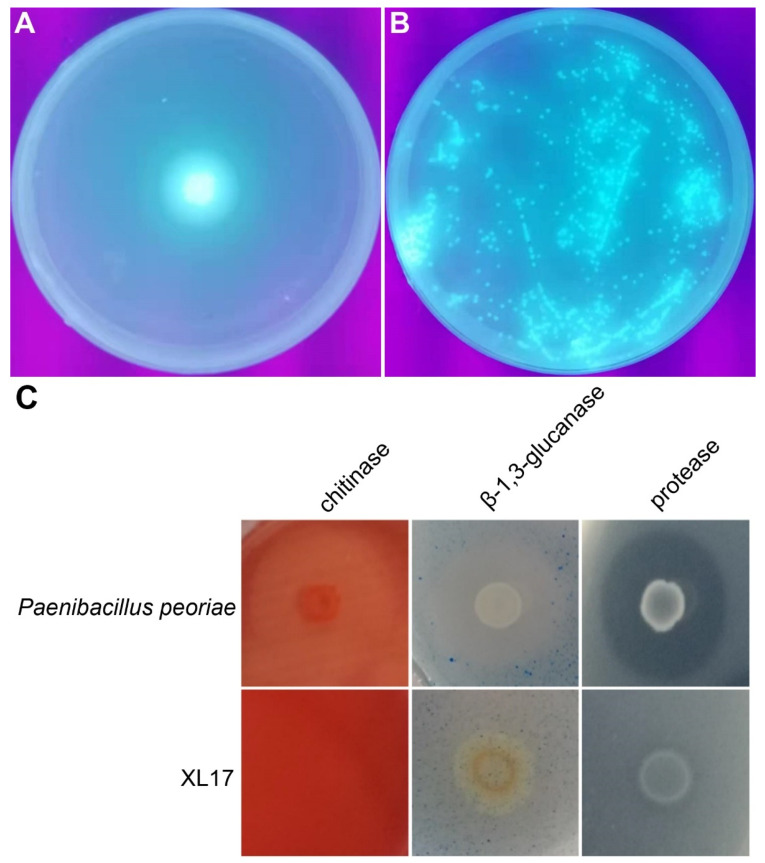
Agar plate assays show bacterial production of fluorescent pyoverdine siderophore and hydrolysis of chitin, β-1,3-glucan, and casein by bacterial chitinase, β-1,3-glucanase, and protease. (**A**) XL17 cells dropped at the center of the King’s B agar plate grow and secrete fluorescent pyoverdine siderophore appearing under UV light. (**B**) XL17 cells spread on the Kings’ B agar plate grow to colonies showing fluorescence. (**C**) *Paenibacillus peoriae* CGMCC 1.3761^T^ used as positive control hydrolyses chitin, β-1,3-glucan, and casein while strain XL17 weakly hydrolyses β-1,3-glucan and casein.

Strain XL17 produced and secreted fluorescent pyoverdine siderophore on the King’s B medium (Figure 9A,B). Strain XL17 showed much weaker β-1, 3-glucanase activity and protease activity compared to *Paenibacillus peoriae* CGMCC 1.3761 and did not show chitinase activity (Figure 9C).

### 3.9. Strain XL17 Produced IAA and ACC Deaminase

**Figure 10:**
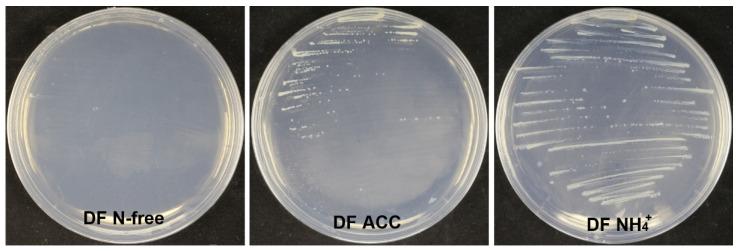
Growing on the DF medium with ACC (DF ACC) as the sole nitrogen source indicates that strain XL17 produces ACC deaminase. Strain XL17 cannot grow on the nitrogen-free DF medium (DF N-free) but grows well on the DF medium with ACC or ammonia (DF NH_4_^+^) as the sole nitrogen source.

Strain XL17 produced about 37 μg of IAA from LB culture at 1 × 10^9^ CFU mL^−1^. Strain XL17 did not grow on the nitrogen-free DF medium but grew well on the DF medium with ACC or ammonia as the sole nitrogen source (Figure 10). In congruence with the presence of the ACC deaminase gene, strain XL17 can produce ACC deaminase.

### 3.10. Strain XL17 and XL17 CF Were Not Toxic to Rice Seeds and Seedlings

To the test toxic or beneficial potentials on plants, rice seeds were inoculated with strain XL17 or treated with XL17 CF, streptomycin, or difenoconazole. XL17 and its CF had no effect on rice seed germination; streptomycin and difenoconazole slightly but not significantly inhibited seed germination. XL17 and its CF significantly increased rice shoot height and dry weight whereas streptomycin significantly reduced rice root length, shoot height, and dry weight, and difenoconazole significantly reduced root length and dry weight (Table 1).

Plant colonization by strain XL17 was determined from the rice seedlings under the gnotobiotic condition. At 7 d after inoculation to rice seeds, strain XL17 was recovered from rhizoplane and surface-sterilized roots and stems. The population of strain XL17 at rhizoplane, in roots, and in stems was 5.0 × 10^8^ ± 5.8 × 10^7^, 7.0 × 10^4^ ± 2.8 × 10^3^, and 2.6 × 10^4^ ± 4.4 × 10^3^ CFU g^−1^ fresh weight, respectively. Strain XL17 can live on and in plants.

## 4. Discussion

The *P. fluorescens* species complex is the most diverse complex within the genus *Pseudomonas* and consists of nine distinct phylogenomic subgroups: *P. fluorescens*, *P. gesardii*, *P. fragi*, *P. mandelii*, *P. jessenii*, *P. koreensis*, *P. chlororaphis*, *P. protegens*, and *P. corrugata* [62,63]. The *P. corrugata* subgroup contains both plant pathogens (such as *P. corrugata* and *P. mediterranea*) and plant growth-promoting bacteria (such as *P. brassicacearum*) [2,64]. Based on recent studies [1,2], our genomic analyses show that the *P. corrugata* subgroup contains at least 31 genomospecies.

We identified that the strain XL17 against Psa and *B. cinerea* belongs to *P. bijieensis* within the *P. corrugata* subgroup and the well-studied DAPG-producing strain 2P24 previously classified into species *P. fluorescens* [54,55,65,66] belongs to a novel genomospecies mostly closely-related to *P. bijieensis*. *P. bijieensis* is a recently identified novel species based on the antifungal strain L22-9^T^ isolated from cornfield soil [67]. A previous functional study about *P. bijieensis* was on the previously misclassified *P. fluorescens* strain NBC275 (=Pf275), which was isolated from riverside soil and showed antifungal activity against plant pathogens *B. cinerea*, *Alternaria solani*, and *Rhizoctonia solani* and insect pathogens *Metarhizium anisopliae* and *Beauveria bassiana* [47]. A mutation generated by transposon in *phlD* completely abolished the antifungal activity of strain Pf275 [68]. Likewise, site-directed mutagenesis in *phlD* completely abolished DAPG production from strain 2P24 and its antagonistic activities against *R. solani* and bacterial pathogen *Ralstonia solanacearum* [65]. The amino acid sequences of PhlD (type III polyketide synthase) (accession no. WP_109753238) of all known *P. bijieensis* strains (L22-9^T^, XL17, Pf275, DR133, St290, and 43MFCvi1.1) are identical. We detected DAPG from XL17 CF showing the antimicrobial activities against *B. cinerea* and Psa. Collectively, these data indicate that DAPG is the key antifungal metabolite produced by *P. bijieensis* and strain 2P24.

DAPG-producing (*phl*+) pseudomonads generally show higher plant-protecting activities than those of *phl*− biocontrol pseudomonads [69]. The *phl* operon for DAPG biosynthesis is mainly present in the genomes of some members of the *P. corrugata* subgroup and the *P. protegens* subgroup within the *P. fluorescens* species complex [70,71]. Melnyk et al. [51] revealed that the LPQ island can serve as a genetic marker for the plant-pathogenic life style of the *P. corrugata* subgroup and is associated with the presence of two small (<10 kb) genetic clusters with unknown functions (putative pathogenicity islets I and II) and the absence of the *phl* operon, Hrp1 T3SS, and the single “orphaned” T3SS effector gene *hopAA*. The phylogeny of the LPQ island [51] and *phlD* (Figure 7) is congruent with the species phylogeny (Figure 6) of the *P. corrugata* subgroup, in which the presence of the *phl* operon and the presence of the LPQ island are generally not overlapping except that genomospecies 25 and 28 contain both clusters (Figure 6). It is likely that the presence of the *phl* operon and the absence of the LPQ island can serve as the genetic marker for the plant-protection life style of the *P. corrugata* subgroup. Therefore, nine genomospecies within Clade 2 of the *P. corrugata* subgroup, i.e., *P. bijieensis* and strain 2P24-represented genomospecies 17, *P. brassicacearum*, *P. kilonensis*, *P. thivervalensis*, *“P. ogarae* subsp. *ogarae*”, and genomospecies 8, 13, and 24 have a high probability to be effective biocontrol agents against plant pathogens.

Among the 11 genomospecies containing the *phl* operon, genomospecies 25 and 28 contain the LPQ island but no Hrp1 T3SS (Figure 6). Interestingly, the presence of the *phl* operon and the absence of the LPQ island happen to be in the presence of Hrp1 T3SS. That is, the presence of Hrp1 T3SS can serve as the genetic marker for the plant-protection life style of the *P. corrugata* subgroup. However, Hrp1 T3SS may not contribute to the known biocontrol activity. The deletion of *rscC* from the Hrp1 T3SS in strain 2P24 did not reduce the production of DAPG, HCN, and siderophores from strain 2P24 and its antagonistic ability against plant pathogens [54]. The function of the Hrp1 T3SS in the *P. corrugata* subgroup and the association between Hrp1 T3SS and the plant-commensal life style of the *P. corrugata* subgroup need further investigation.

DAPG is not the only antimicrobial metabolite from *P. bijieensis* and strain 2P24 contributing to their biocontrol activities. A mutation in *P. bijieensis* Pf275 and strain 2P24 did not abolish their biocontrol activities [47,65]. The gene clusters responsible for the biosynthesis of the pyoverdine siderophore, the linear lipopeptide (Val-Ala-Gln-Ala-Val-Ala-Pro-Thr), and the aryl polyene compound were also identified for their contribution to the biocontrol activity of *P. bijieensis* Pf275 [47,72]. These gene clusters are also present in the genome of the other *P. bijieensis* strains and strain 2P24 and may also contribute to their biocontrol activities. Although XL17 produced pyoverdine siderophores on King’s B medium, siderophore production may not contribute to the antimicrobial activities detected on the iron sufficient PDA and LB media.

Cyclic lipopeptides produced by plant-associated pathogenic and beneficial *Pseudomonas* are biosurfactants involved in motility, surface attachment, biofilm regulation, virulence, antimicrobial activities, and the induction of plant resistance [73,74]. Although the biosynthetic gene clusters for cyclic lipopeptides were not clearly identified in *P. bijieensis*, viscosin- and orfamide-like lipopeptides were detected by MALDI-TOF-MS from XL17 cells. Viscosins show broad-spectrum antimicrobial activities against fungi, Gram-positive bacteria, and Gram-negative bacteria but not *Pseudomonas* pathogens [73]. Orfamides show antifungal activities but no antibacterial activities [73]. Cyclic lipopeptides of the viscosin family and orfamide family may participate in XL17 against *B. cinerea*.

Our genomic analyses and metabolic analyses on strain XL17 and previous studies on potent biocontrol pseudomonads [69,73,74], including strains Pf275 and 2P24 [65,68], strongly suggest that DAPG and lipopeptides are the key antimicrobial metabolites from strain XL17 to breach the cell envelopes of Psa and *B. cinerea*. On the other hand, DAPG and cyclic lipopeptides produced by plant-associated pseudomonads [73,74,75,76,77] may be toxic to plants [74,78]. Thus, we did a phytotoxic test and demonstrated that strain XL17 and its CF were not toxic to rice seeds and seedlings but increased rice shoot height and dry weight. Therefore, XL17 and its CF can promote plant growth. The bacterial production of IAA and ACC deaminase may be involved in this plant growth-promotion [79].

Strain XL17 showed no chitinase activity and much weaker β-1, 3-glucanase and protease activities compared with *Paenibacillus peoriae* CGMCC 1.3761^T^, which showed strong hydrolytic enzyme activities but no antagonistic activity against *B. cinerea* [24]. It is likely that hydrolytic enzymes from strain XL17 may not play an important role in breaching the cell envelope of *B. cinerea*.

Our initial intention was to screen biocontrol endophytes, which are competent plant colonizers and adapt to plant immune response to form a mutualistic association with plants [24]; we found *P. bijieensis* XL17 from a surface-sterilized rape crown gall. However, *P. bijieensis* L22-9^T^ and Pf275 and the closely related strain 2P24, which also show potent biocontrol activities, were isolated from soil [65,67,68]. Our genomic analyses and previous molecular studies on strains Pf275 and 2P24 [65,68] revealed that genetic lineage with the conserved *phl* operon and NRPS gene clusters rather than the original source is the key to find potent biocontrol agents. Most *phlD*-carrying strains within Clade 2 of the *P. corrugata* subgroup (Figure 6) are potential biocontrol agents. Lipopeptide families also determine the plant-pathogenic or plant-protecting behavior of the strains within the *P. corrugata* subgroup [73,74]. Therefore, we propose a rapid approach to identify effective biocontrol pseudomonads belonging to the *P. corrugata* subgroup after screening out a potent antimicrobial bacterium. First, the phylogenetic analysis of nearly complete 16S rRNA gene sequences identifies an antimicrobial strain within the *P. corrugata* subgroup. Second, PCR amplification and sequencing of the *phlD* sequence identifies a DAPG producer. Third, MALDI-TOF-MS identifies a lipopeptide producer.

## 5. Conclusions

Our phenotypic, genomic, and metabolic analyses identified that *P. bijieensis* XL17 producing DAPG and lipopeptides are able to control bacterial canker and gray mold pathogens of kiwifruit. The conserved presence of the *phl* operon for DAPG biosynthesis and the NRPS gene clusters for lipopeptide biosynthesis other than the LPQ island determines the biocontrol strength of *P. bijieensis* and its relatives within the *P. corrugata* subgroup against fungal and bacterial phytopathogens.

## Figures and Tables

**Table 1:** Effects of strain XL17 and its culture filtrate (CF) on rice seed germination and growth.

Treatment	Germination (%)	Root Length (mm)	Shoot Height (mm)	Dry Weight (mg)
Control	100 ± 0.0 ^a^*	45.6 ± 0.0 ^ab^	48.2 ± 0.0 ^b^	6.8 ± 0.1 ^b^
XL17	100 ± 0.0 ^a^	45.9 ± 0.0 ^a^	50.8 ± 0.0 ^a^	7.4 ± 0.1 ^a^
Streptomycin	98.3 ± 1.7 ^a^	38.9 ± 0.0 ^d^	42.0 ± 0.1 ^c^	3.9 ± 0.1 ^d^
Difenoconazole	98.3 ± 1.7 ^a^	42.1 ± 0.1 ^c^	49.5 ± 0.0 ^ab^	6.3 ± 0.2 ^c^
10% CF	100 ± 0.0 ^a^	44.5 ± 0.0 ^ab^	49.9 ± 0.1 ^a^	7.2 ± 0.1 ^a^
15% CF	100 ± 0.0 ^a^	44.3 ± 0.0 ^b^	50.1 ± 0.1 ^a^	7.3 ± 0.2 ^a^
20% CF	100 ± 0.0 ^a^	44.4 ± 0.1 ^b^	50.5 ± 0.0 ^a^	7.2 ± 0.0 ^a^

* Values are mean ± standard error of three replicates for each treatment. Different letters show significant difference at *p* < 0.05.

## Data Availability

Not applicable.

## Supplementary Materials

The following supporting information can be downloaded at: https://www.mdpi.com/article/10.3390/microorganisms10020425/s1, Figure S1: Compact view of gene clusters for biosynthesis of secondary metabolites identified by antiSMASH version 6.0 (https://antismash.secondarymetabolites.org/, accessed on 10 October 2021) using relaxed detection strictness from whole genome sequences of *Pseudomonas bijieensis* strains and the neighboring genomospecies strain *Pseudomonas* sp. 2P24.; Table S1: Digital DNA-DNA hybridization (dDDH) values between genome pairs of strains within *Pseudomonas corrugata* subgroup; Table S2: Digital DNA-DNA hybridization (dDDH) values between genome pairs of strains belonging to “*Pseudomonas ogarae*”.
