# Supplementary material for: Pseudomonas bijieensis Strain XL17 within the P. corrugata Subgroup Producing 2,4-Diacetylphloroglucinol and Lipopeptides Controls Bacterial Canker and Gray Mold Pathogens of Kiwifruit
Source: Microorganisms. 2022 Feb 12;10(2):425. doi: 10.3390/microorganisms10020425 (PMC8878242; doi:10.3390/microorganisms10020425)
Supplement: Supplementary file 1 [file microorganisms-10-00425-s001.zip › microorganisms-1533627-supplementary/Supplementary material/Figure S1.pdf]

Pseudomonas bijieensis XL17 (JAJQKS000000000.1) 17 regions

Region	Type	From	To	Most similar known cluster		Similarity
Region 1.1	NRPS	1	38,341	pyoverdin	NRP	11%
Region 2.1	RiPP-like	161,167	170,284			
Region 2.2	RiPP-like	868,157	878,981			
Region 2.3	betalactone	892,282	915,516	fengycin	NRP	13%
Region 3.1	NRPS	426,734	479,720	pyoverdin	NRP	10%
Region 3.2	NAGGN	605,603	620,493			
Region 4.1	RiPP-like	576,980	587,849			
Region 5.1	NRPS	116,926	182,967	entolysin	NRP	8%
Region 5.2	butyrolactone	604,105	617,524			
Region 5.3	NRPS	628,486	668,230	crochelin A	NRP + Polyketide	7%
Region 7.1	arylpolyene	265,837	309,448	APE Vf	Other	40%
Region 10.1	NRPS	1	36,186			
Region 10.2	T3PKS	85,829	126,917	2,4-diacetylphloroglucinol	Polyketide	100%
Region 13.1	lanthipeptide-class-ii	85,406	108,480			
Region 14.1	redox-cofactor	27,809	49,974	lankacidin C	NRP + Polyketide	13%
Region 17.1	NRPS-like	28,708	64,947	fragin	NRP	37%
Region 20.1	NRPS	1	29,241	pyoverdin	NRP	10%

Pseudomonas bijieensis L22-9[T] (CP048810.1) 15 regions

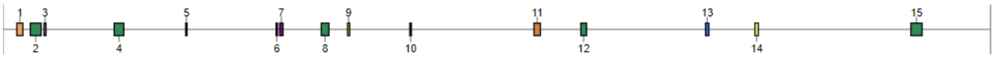

Region	Type	From	To	Most similar known cluster		Similarity
Region 1	T3PKS	94,261	135,310	2,4-diacetylphloroglucinol	Polyketide	100%
Region 2	NRPS	185,776	259,864	crochelin A	NRP + Polyketide	7%
Region 3	butyrolactone	279,602	293,021			
Region 4	NRPS	757,542	823,598	syringomycin	NRP	17%
Region 5	RiPP-like	1,245,173	1,254,394			
Region 6	RiPP-like	1,861,034	1,870,319			
Region 7	betalactone	1,885,193	1,908,427	fengycin	NRP	13%
Region 8	NRPS	2,168,914	2,221,105	pyoverdin	NRP	10%
Region 9	NAGGN	2,348,114	2,363,004			
Region 10	RiPP-like	2,772,377	2,783,246			
Region 11	arylpolyene	3,618,892	3,662,503	APE Vf	Other	40%
Region 12	NRPS-like	3,938,199	3,976,983	fragin	NRP	37%
Region 13	redox-cofactor	4,788,138	4,810,303	lankacidin C	NRP + Polyketide	13%
Region 14	lanthipeptide-class-ii	5,124,605	5,147,679			
Region 15	NRPS	6,187,494	6,263,594	pyoverdin	NRP	19%

Pseudomonas bijieensis Pf275 (CP031648.1) 15 regions

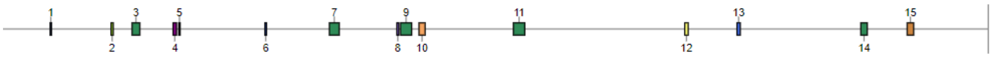

Region	Type	From	To	Most similar known cluster		Similarity
Region 1	RiPP-like	322,774	331,524			
Region 2	NAGGN	730,798	745,688			
Region 3	NRPS	871,654	923,725	pyoverdin	NRP	10%
Region 4	betalactone	1,146,428	1,169,662	fengycin	NRP	13%
Region 5	RiPP-like	1,184,534	1,193,819			
Region 6	RiPP-like	1,762,091	1,772,936			
Region 7	NRPS	2,194,062	2,260,115	entolysin	NRP	8%
Region 8	butyrolactone	2,646,084	2,659,503			
Region 9	NRPS	2,670,678	2,745,704	crochelin A	NRP + Polyketide	7%
Region 10	T3PKS	2,794,756	2,835,805	2,4-diacetylphloroglucinol	Polyketide	100%
Region 11	NRPS	3,427,563	3,503,750	pyoverdin	NRP	18%
Region 12	lanthipeptide-class-ii	4,576,049	4,599,123			
Region 13	redox-cofactor	4,926,002	4,948,167	lankacidin C	NRP + Polyketide	13%
Region 14	NRPS-like	5,755,901	5,799,431	fragin	NRP	37%
Region 15	arylpolyene	6,066,772	6,110,383	APE Vf	Other	40%

***Pseudomonas bijieensis* DR133 (CP048607.1) 16 regions**

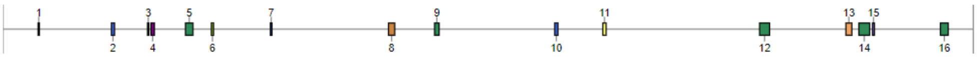

Region	Type	From	To	Most similar known cluster		Similarity
Region 1.1	RiPP-like	245,162	253,923			
Region 1.2	ranthipeptide	756,554	777,984	pyoverdin	NRP	8%
Region 1.3	RiPP-like	1,008,348	1,017,632			
Region 1.4	betalactone	1,032,502	1,055,736	fengycin	NRP	13%
Region 1.5	NRPS	1,272,733	1,324,834	pyoverdin	NRP	10%
Region 1.6	NAGGN	1,453,510	1,468,400			
Region 1.7	RiPP-like	1,864,933	1,875,802			
Region 1.8	arylpolyene	2,688,468	2,732,079	APE Vf	Other	40%
Region 1.9	NRPS-like	3,010,000	3,041,504	fragin	NRP	37%
Region 1.10	redox-cofactor	3,847,813	3,869,978	lankacidin C	NRP + Polyketide	13%
Region 1.11	lanthipeptide-class-ii	4,183,262	4,206,336			
Region 1.12	NRPS	5,276,413	5,346,362	pyoverdin	NRP	19%
Region 1.13	T3PKS	5,878,549	5,919,598	2,4-diacetylphloroglucinol	Polyketide	100%
Region 1.14	NRPS	5,968,688	6,043,948	crochelin A	NRP + Polyketide	7%
Region 1.15	butyrolactone	6,063,932	6,077,351			
Region 1.16	NRPS	6,537,288	6,591,672			

***Pseudomonas bijieensis* St290 (AP014703.1) 17 regions**

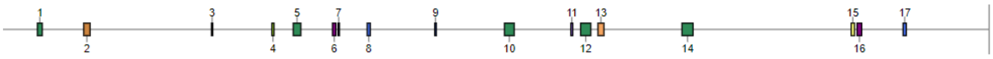

Region	Type	From	To	Most similar known cluster		Similarity
Region 1	NRPS-like	243,107	276,241	fragin	NRP	37%
Region 2	arylpolyene	560,905	604,516	APE Vf	Other	40%
Region 3	RiPP-like	1,437,793	1,446,538			
Region 4	NAGGN	1,852,340	1,867,230			
Region 5	NRPS	1,999,040	2,051,164	pyoverdin	NRP	10%
Region 6	betalactone	2,268,644	2,291,878	fengycin	NRP	13%
Region 7	RiPP-like	2,306,622	2,315,907			
Region 8	redox-cofactor	2,506,526	2,528,070			
Region 9	RiPP-like	2,970,190	2,981,035			
Region 10	NRPS	3,448,845	3,515,682	syringomycin	NRP	17%
Region 11	butyrolactone	3,904,593	3,918,012			
Region 12	NRPS	3,971,736	4,041,547	crochelin A	NRP + Polyketide	7%
Region 13	T3PKS	4,090,689	4,131,738	2,4-diacetylphloroglucinol	Polyketide	100%
Region 14	NRPS	4,667,485	4,743,585	pyoverdin	NRP	19%
Region 15	lanthipeptide-class-ii	5,828,841	5,851,915			
Region 16	terpene	5,869,669	5,901,378	oxalomycin B	NRP + Polyketide	6%
Region 17	redox-cofactor	6,185,216	6,207,381	lankacidin C	NRP + Polyketide	13%

***Pseudomonas bijieensis* 43MFCvi1.1 (QGDM00000000.1) 18 regions**

Region	Type	From	To	Most similar known cluster		Similarity
Region 1.1	NAGGN	101,486	116,376			
Region 1.2	NRPS	241,767	294,765	pyoverdin	NRP	10%
Region 1.3	betalactone	511,731	534,965	fengycin	NRP	13%
Region 1.4	RiPP-like	548,515	559,231			
Region 2.1	arylpolyene	271,121	314,732	APE Vf	Other	40%
Region 4.1	butyrolactone	180,262	193,681			
Region 4.2	NRPS	203,563	279,366	crochelin A	NRP + Polyketide	7%
Region 4.3	T3PKS	328,046	369,095	2,4-diacetylphloroglucinol	Polyketide	100%
Region 7.1	NRPS	79,965	147,371	syringomycin	NRP	17%
Region 9.1	NRPS	1	67,768	pyoverdin	NRP	19%
Region 11.1	redox-cofactor	59,907	82,072	lankacidin C	NRP + Polyketide	13%
Region 15.1	RiPP-like	76,494	87,363			
Region 18.1	RiPP-like	124,294	135,139			
Region 19.1	terpene	13,538	45,160	oxalomycin B	NRP + Polyketide	6%
Region 20.1	lanthipeptide-class-ii	85,331	108,405			
Region 26.1	ranthipeptide	3,287	24,717	pyoverdin	NRP	8%
Region 28.1	NRPS-like	1	27,396	fragin	NRP	25%
Region 32.1	NRPS	1	2,738	rhizomide A / rhizomide B / rhizomide C	NRP	100%

***Pseudomonas* sp. 2P24 (CP025542.1) 14 regions**

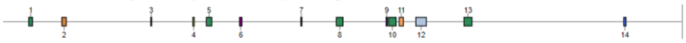

Region	Type	From	To	Most similar known cluster		Similarity
Region 1	NRPS-like	256,825	294,469	fragin	NRP	37%
Region 2	arylpolyene	578,132	621,743	APE Vf	Other	40%
Region 3	RiPP-like	1,443,160	1,451,950			
Region 4	NAGGN	1,851,929	1,866,663			
Region 5	NRPS	1,983,759	2,036,400	pyoverdin	NRP	11%
Region 6	betalactone	2,306,211	2,329,529	fengycin	NRP	13%
Region 7	RiPP-like	2,902,179	2,913,024			
Region 8	NRPS	3,247,100	3,312,791	syringomycin	NRP	23%
Region 9	butyrolactone	3,730,422	3,743,841			
Region 10	NRPS	3,754,217	3,828,993	crochelin A	NRP + Polyketide	7%
Region 11	T3PKS	3,858,216	3,899,265	2,4-diacetylphloroglucinol	Polyketide	100%
Region 12	hserlactone , PKS-like , transAT-PKS	4,019,699	4,122,551	pseudomonic acid A	Polyketide:Iterative type I + Polyketide:Trans-AT type I	100%
Region 13	NRPS	4,488,687	4,565,796	pyoverdin	NRP	20%
Region 14	redox-cofactor	6,041,763	6,063,928	lankacidin C	NRP + Polyketide	13%

Figure S1. Compact view of gene clusters for biosynthesis of secondary metabolites identified by antiSMASH version 6.0 (https://antismash.secondarymetabolites.org/) using relaxed detection strictness from whole genome sequences of *Pseudomonas bijieensis* strains and the neighboring genomospecies strain *Pseudomonas* sp. 2P24.
